# An extracellular humanized IFNAR immunocompetent mouse model for analyses of human interferon alpha and subtypes

**DOI:** 10.1080/22221751.2023.2287681

**Published:** 2023-11-23

**Authors:** Yumeng Li, Asha Ashuo, Menghan Hao, Yaming Li, Jianyu Ye, Jiangxia Liu, Ting Hua, Zhong Fang, Jianhua Li, Zhenghong Yuan, Jieliang Chen

**Affiliations:** a Key Laboratory of Medical Molecular Virology (MOE/NHC), Research Unit of Cure of Chronic Hepatitis B Virus Infection (CAMS), Shanghai Frontiers Science Center of Pathogenic Microbes and Infection, School of Basic Medical Sciences, Shanghai Medical College Fudan University, Shanghai, People’s Republic of China; b Shanghai Institute of Infectious Disease and Biosecurity, Shanghai, People’s Republic of China

**Keywords:** Interferon (IFN), interferon alpha/beta receptor 1 (IFNAR1), interferon alpha/beta receptor 2 (IFNAR2), humanized mouse model, hepatitis B virus (HBV), JAK/STAT signal pathway (JAK-STAT)

## Abstract

Type I interferons (IFN-Is) have key roles in immune defense and treatments for various diseases, including chronic hepatitis B virus (HBV) infection. All IFN-Is signal through a shared IFN-I heterodimeric receptor complex comprising IFN-α receptor 1 (IFNAR1) and IFNAR2 subunits, but differences in antiviral and immunomodulatory responses among IFN-I subtypes remain largely unknown. Because the IFN-IFNAR interactions are species-specific, mice exhibit weak responses to human IFN-I. To more fully characterize the actions of human IFN-α and its subtypes *in vivo*, a gene targeting strategy was employed to generate gene knock-in mice with extracellular-humanized IFNAR1/2 (IFNAR-hEC) in the C57BL/6N strain. IFNAR-hEC mice actively responded to human IFN-I, and endogenous mouse IFN-I signalling remained active in heterozygous mice (*Ifnar*^hEC/+^). Analyses of IFNAR-hEC mice and isolated cells showed that human IFN-α2 and α14 subtypes exerted differential effect on the activation of JAK-STAT signalling and immune responses. Compared with IFN-α2, IFN-α14 induced greater activation of STAT1/2 and IFN-stimulated genes, synergistically elicited IFN-α and -γ signalling, and induced higher numbers of antigen-specific CD8^+^ T cells. Moreover, IFNAR-hEC mice with HBV replication displayed long-term viral suppression upon treatment with the clinically-used PEGylated hIFN-α2. These results indicate that IFNAR-hEC mice may be useful for elucidating antiviral and immunomodulatory functions of human IFN-Is and for conducting preclinical studies. A better understanding of the distinct activities of IFN-α subtypes can provide insights concerning the development of improved IFN-based therapy.

## Introduction

Interferons (IFNs), a group of cytokines first described in 1957 [1], have key roles in immune responses. Because of their potent antiviral and antitumour activities, IFNs are utilized in the treatment of viral infections and cancers. More than 20 distinct IFN genes have been identified in humans, and they are generally classified into three types according to receptor usage: type I IFNs (IFN-Is; e.g. IFN-α and IFN-β), type II IFN (IFN-γ), and type III IFN (IFN-λ) [2, 3]. Among these, IFN-α is the largest type; it is further divided into 13 subtypes, all located on chromosome 9. IFN-α signals through a shared IFN-I heterodimeric receptor complex that consists of IFN-α receptor 1 (IFNAR1) and IFNAR2 subunits. The binding of IFN-α to IFNAR1/2 initiates the JAK-STAT signalling pathway, leading to the expression of interferon-stimulated genes (ISGs). ISGs encompass a diverse group of proteins that serve as effectors in efforts to control viral infections and regulate immune responses [4].

A longstanding question in IFN research is why there are so many IFN-α subtypes that appear to differ in biological functions, although all of which signal through a common receptor: IFNAR1/2 [5]. Our recent findings regarding the distinct antiviral effects of IFN-α subtypes, particularly involving hepatitis B virus (HBV), have advanced the overall understanding of this issue [6]. IFN-α2(a/b), the early-discovered and most-studied subtype, has been approved for treatment of chronic hepatitis B since the 1990s [7]. In contrast to the nucleot(s)ide analogues (NAs) that target HBV reverse transcription to inhibit viral replication, IFN-α restricts HBV by affecting multiple stages of the viral life cycle. Thus, it has unique advantages for curing HBV infection, including a finite course of treatment, relatively higher rates of HBeAg and HBsAg seroconversion, and a lower incidence of hepatocellular carcinoma (HCC) [8, 9]. However, the proportion of patients that respond to current IFN therapy remains low (20∼40%), hindering its clinical application [10]. We have previously found that human IFN-α subtype 14 is the most effective subtype against HBV in cell models and immunodeficient human-liver chimeric mice [6]. It remains unclear how IFN-α14 activates antiviral signalling and functions in immunocompetent settings.

Because IFN-IFNAR interactions are species-specific, mice barely respond to human IFN-I [11]. To more fully characterize the action of human IFN-α and its subtypes *in vivo*, we used a gene targeting strategy to generate gene knock-in mice with extracellular humanized IFNAR1/2 (IFNAR-hEC) in the C57BL/6N strain. We observed that IFNAR-hEC mice showed active responses to human IFN-I, while maintaining intact endogenous mouse IFN-I signalling in the heterozygous mice. When using IFNAR-hEC mice and isolated cells from these mice, we found that, in comparison to human IFN-α2, IFN-α14 synergistically induced IFN-α and -γ signalling and exhibited enhanced immunomodulatory potential. Furthermore, IFNAR-hEC mice with HBV replication displayed long-term viral suppression when treated with PEGylated hIFN-α2 (PEGASYS), a widely used therapy in clinical practice. These results indicate that IFNAR-hEC mice may be useful for elucidating antiviral and immunomodulatory functions of human IFN-I and for conducting preclinical studies. A deeper understanding of the distinct activities of IFN-α subtypes can shed light on the development of improved IFN-based therapy.

## Materials and methods

### Generation of human IFNAR gene knock-in mice

IFNAR-hEC mice (C57BL/6N mouse strain) were generated using TurboKnockout ES cell targeting technique provided by Cyagen Biosciences (Suzhou, China) Inc. The humanized receptor coding sequence was constructed by fusing the extracellular domain of human IFNAR with the transmembrane and cytoplasmic segments of the mouse receptor. The mouse *Ifnar1* gene (GenBank accession number: NM_010508.2; Ensembl: ENSMUSG00000022967) and mouse *Ifnar2* gene (GenBank accession number: NM_010509.2; Ensembl: ENSMUSG00000022971) are both located on mouse chromosome 16. Two specific regions were selected for targeting: the first region spanned from the ATG start codon (aa.27) in exon 2 to a partial intron 2 of mouse *Ifnar1*, and the second region spanned from the ATG start codon (aa.22) in exon 3 to a partial intron 3 of mouse *Ifnar2*. The regulated and signal sequences of mouse *Ifnar* were retained. The humanized receptor coding gene was amplified and incorporated into a targeting vector with resistance gene flanked by a pair of homology arms. Electroporation of the *Ifnar1* targeting vector into ES cells was followed by G418 selection (200 μg/mL) 24 h post-electroporation. The X-Porator H1 electroporator (Etta Biotech Co., Suzhou) was utilized for electroporation. A total of 188 G418-resistant clones were picked and screened positive for the targeted integration of the gene using PCR, which was further confirmed by Southern blot analysis. The linearized *Ifnar2* targeting vector was then transfected into ES cell clones obtained from the previous construct. These transfected ES cells were subjected to puromycin selection (0.8 μg/mL) 24 h post-electroporation, and double-positive clones were expanded and identified as described previously. The targeted ES cells were microinjected into host blastocysts and subsequently transferred into surrogate mothers. Genotyping of founders was performed using PCR, and they were crossed with wildtype mice. F1 founders were identified by PCR and bred to obtain homozygous mice for germline transmission testing. Genotyping of the *Ifnar1* knock-in allele was conducted using the following primer pairs: forward, CCGTACTGGTCATTACTGTGGTT; reverse R1, ACCAAATGCTTCCCACATTAAAAGGA; reverse R2, CACTGAACTTGAAAGGTCATGTTTGC with an annealing temperature of 60 °C (band sizes are 859 bp for homozygous, 859 and 346 bp for heterozygous, and 346 bp for wild-type). Genotyping of the *Ifnar2* knock-in allele was performed using the following primer pairs: forward, CCACATTACCCAAGAGCATCCATAC; reverse R1, CCTCTACCTAGAAAGGATTCCAATAAACTG; reverse R2, ATTGTGTGAGCAACTGAACAACGT with an annealing temperature of 60 °C (band sizes are 470 bp for homozygous, 470 and 345 bp for heterozygous, and 345 bp for wild-type).

### Recombinant IFN-α proteins

The recombinant IFN-α proteins for cell culture were purchased from PBL Assay Science (Piscataway, NJ) and listed in Table S3. The recombinant IFN-α proteins used in mice were prepared as described [12]. The plasmids encoding for IFN-α2 and IFN-α14 were synthesized by GeneScript and were transformed into competent BL21 *Escherichia coli* cells for protein expression. All the recombinant IFNs were confirmed to be endotoxin-free (<0.1 endotoxin unit per microgram; Shanghai Labway Clinical Laboratory, Shanghai, China).

### Isolation of primary mouse hepatocytes (PMHs)

Mice were euthanized and perfused through the inferior vena cava with perfusion buffer to chelate calcium and wash out blood, then perfused with digestion buffer (type IV collagenase in DMEM medium); all buffers were prewarmed to 37°C. Immediately after digestion, the liver was gently dissected and transferred to a 6 cm cell plate containing DMEM, it was then ruptured with forceps and the contents were filtered through a 100 µm cell strainer. Next, the cell pellet was resuspended with 90% Percoll solution and centrifuged at 700 × g for 10 min to remove dead cells and debris. The remaining cells were then washed twice and resuspended in DMEM, then counted and plated on 24-well plates (5 × 10^5^ cells per well) that had been pre-coated with type I collagen (C3867, Sigma).

### In vivo killing assay

Naive lymphocytes from the draining lymph nodes and spleens of wildtype mice were isolated as target cells [13]. Cells pulsed with 5 µg/mL OVA_257-264_ peptide (S7951, Sigma-Aldrich) at 37°C for 1 h were labelled with 5 μM CFSE (CFSE^high^); cells not incubated with peptides were labelled with 0.5 μM CFSE (CFSE^low^). Next, CFSE^high^ and CFSE^low^ cells were mixed in a 1:1 ratio and intravenously transferred into recipient mice (1 × 10^7^ cells per mouse). Approximately 24 h later, splenocytes isolated from recipient mice were analyzed for CFSE staining by flow cytometry, and the percentage of specific killing was calculated as follows: specific killing (%) = 100 – [(CFSE^high^ / CFSE^low^) _immunized_ / (CFSE^high^ / CFSE^low^) _control_] × 100.

### Enzyme-linked immunospot assay (ELISPOT)

IFN-γ ELISPOT assays [14] were performed according to the manufacturer’s instructions (551083, BD Biosciences, USA). The kit included the unlabelled capture antibody, biotinylated detection antibody and enzyme conjugate. Briefly, IFN-γ ELISPOT plates were pre-coated with unlabelled capture antibody at 4°C overnight. After plates had been blocked with RPMI 1640 medium, splenocytes were seeded in triplicate at 5 × 10^5^ cells per well and incubated with 5 µg/mL OVA_257-264_ at 37°C for 24 h. Next, IFN-γ production was analyzed by incubation with biotinylated detection anti-mouse IFN-γ antibody and streptavidin-conjugated HRP. Spots were visualized with AEC substrate (551951, BD Biosciences, USA) and counted using an ELISPOT auto analysis system.

### Statistical analysis

Data were analyzed using GraphPad Prism 8.0 (GraphPad Software Inc.). Statistics analyses were performed using the Student’s *t* test and data were presented as the mean ± standard deviation (SD). A *P* value < 0.05 was considered to be statistically significant.

## Results

### Design and generation of transgenic mice with extracellular humanized IFNAR

**Figure 1.**
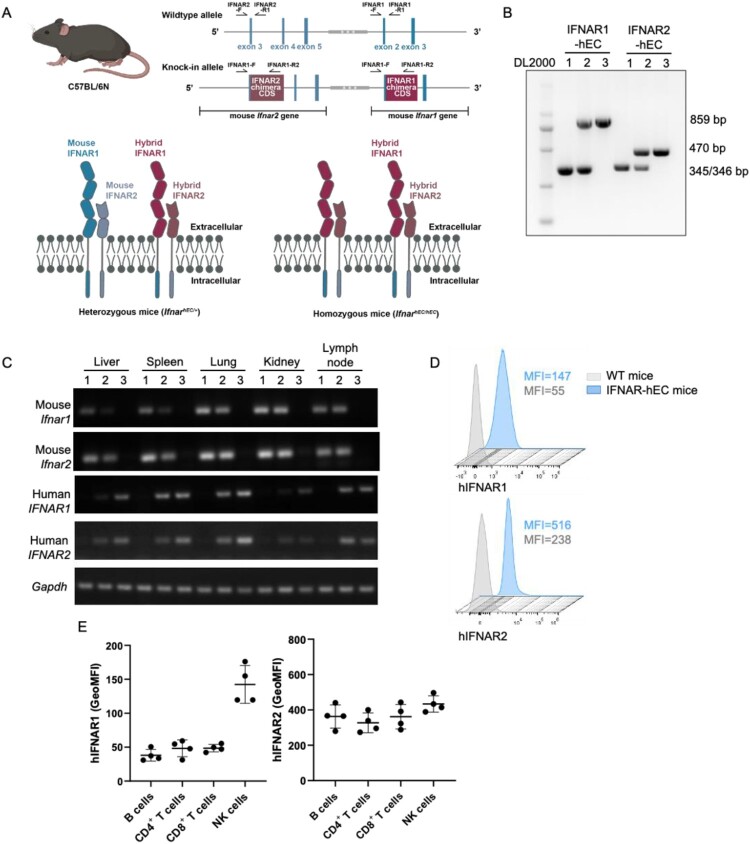
**Generation of IFNAR extracellular-humanized mice**. (A) Schematic representation of the knock-in strategy for the IFNAR-hEC coding gene. Two knock-in vectors were constructed by inserting cDNA encoding the extracellular domain of human IFNAR fused with the mouse transmembrane and cytoplasmic segments into the respective mouse *Ifnar1* and *Ifnar2* genomic loci. Arrows indicate PCR primers for genotyping. (B) A representative result of genotyping to confirm the homozygosity of IFNAR-hEC knock-in alleles. Wild-type alleles were regarded as bands at 345/346 bp; *Ifnar1* and *Ifnar2* knock-in alleles were regarded as bands of 859 and 470 bp, respectively. (C) Representative results of RT-qPCR analysis for the tissue distribution of mouse *Ifnar* and IFNAR-hEC transcriptional level. Numbers above denote the mouse genotypes: (1) *Ifnar^+/+^*, (2) *Ifnar*^hEC/*+*^, (3) *Ifnar*^hEC/hEC^. (D) Flow cytometry analysis for the expression of IFNAR-hEC in *Ifnar*^+/+^ (wild-type) and *Ifnar*^hEC/hEC^ (Ho) by human IFNAR1/2 antibodies. (E) Expression levels of hIFNAR-EC on different lymphocyte subsets in *Ifnar*^hEC/hEC^.

Schematic illustration of the generation of IFNAR-hEC mice was presented in Figure 1A and Fig. S1. The humanized IFNAR1/2 consists of mouse transmembrane and intracellular regions with only the extracellular domain humanized. We identified double-positive targeted embryonic stem cell clones (C57BL/6N mouse strain) and performed microinjections into blastocysts, then transferred those blastocysts into surrogate mothers. Genotype founders were confirmed by genotyped PCR and subsequently bred to obtain homozygous mice. PCR products of 859 and 470 bp, amplified using the primers shown in Figure 1A and Table S2, corresponded to the *Ifnar1* knock-in allele and *Ifnar2* knock-in allele, respectively; PCR products of 345/346 bp indicated the unedited alleles (Figure 1B). RT-qPCR analysis of cDNA for IFNAR-hEC or mouse *Ifnar* from different organs revealed accurate amplification of the specific target in each reaction (Figure 1C). In cDNA samples of homozygotes (*Ifnar*^hEC/hEC^), IFNAR-hEC-specific primers efficiently amplified target sequences, whereas primers specific for the mouse *Ifnar* region did not recognize the target sequences. Similarly, in cDNA samples from wild-type mice (*Ifnar*^+/+^), only *Ifnar* sequence primers facilitated amplification. Both IFNAR-hEC and Ifnar were amplified in heterozygous mice (*Ifnar*^hEC/+^). Flow cytometry staining analyses of peripheral blood mononuclear cells (PBMCs) were performed to confirm the expression of IFNAR-hEC. Compared with wild-type mice, IFNAR-hEC mice displayed a significant increase in signal intensity (Figure 1D). Subsequently, subset-specific antibodies were utilized to assess the expression levels of IFNAR-hEC in lymphocyte subpopulations. The highest receptor expression level of IFNAR-hEC was observed on natural killer cells; CD4^+^ T cells, CD8^+^ T cells and B cells exhibited similar expression levels (Figure 1E). Taken together, our results indicate the successful establishment of an IFANR-hEC mouse model.

### IFNAR-hEC mice actively respond to human IFN-Is and retain an intact endogenous IFN-Is

**Figure 2.**
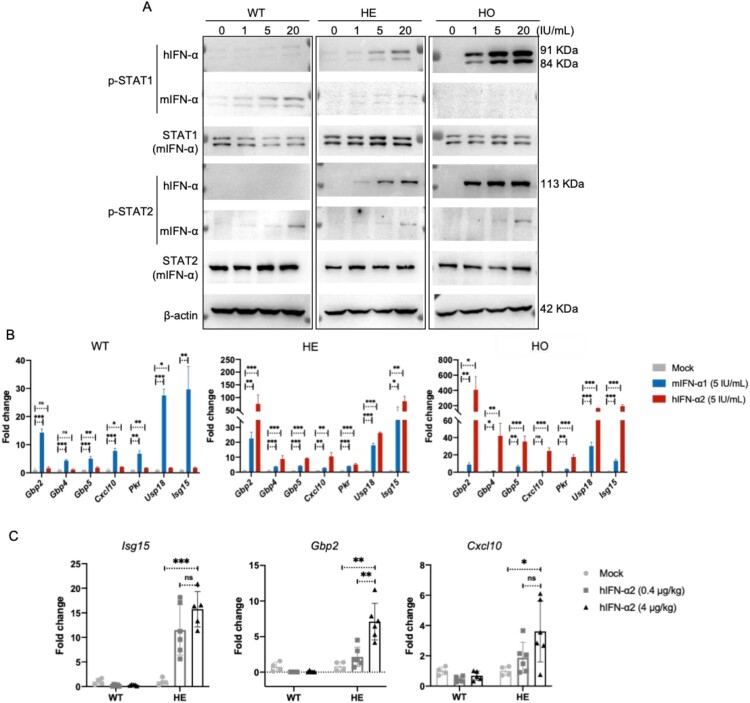
**Responses of IFNAR-hEC mice to treatment with human IFN-I**. (A, B) Primary mouse hepatocytes isolated and treated with human IFN-α2 or mouse IFN-α1 for 0.5 and 6 h were extracted for analysis of p-STAT1(Y701), p-STAT2(Y690), and expression of ISGs, respectively. (C) *Ifnar*^hEC/+^ mice were intravenously administered hIFN-α2 for 6 h, then sacrificed. Total RNA from liver samples was used for RT-qPCR analysis of representative ISGs. Statistically significant differences are indicated by * for *p* < 0.05, ** for *p* < 0.01 and *** for *p* < 0.001. Abbreviations: WT, wild-type mouse; HE, heterozygous IFNAR-hEC mouse; HO, homozygous IFNAR-hEC mouse.

To explore the responses of IFNAR-hEC mice to IFN-I treatment, primary mouse hepatocytes (PMHs) were isolated, then separately treated with gradient concentrations of human and mouse IFN-α. PMHs treated with human IFN-α displayed concentration-dependent phosphorylation of STAT1 and STAT2, along with effective activation of ISGs expression (Figure 2A-B). In contrast, wild-type mice did not respond to human IFN-α. We further validated response *in vivo* by intravenous administration of human IFN-α. RT-qPCR revealed that ISGs were significantly up-regulated in treated mice (Figure 2C). After treatment with human IFN-γ, PMHs from IFNAR-hEC mice did not exhibit activation of the JAK-STAT pathway (Fig. S2A-B). Moreover, signalling activation tests were performed with all 13 human IFN-α subtypes in PMHs from IFNAR-hEC mice, revealing distinct activation patterns across all subtypes (Fig. S2C). After treatment with IFN-β and IFN-ω, PMHs also exhibited active responses (Fig. S2D-E). These results suggested that IFNAR-hEC mice display a specific and robust responsiveness to human IFN-Is.

**Figure 3.**
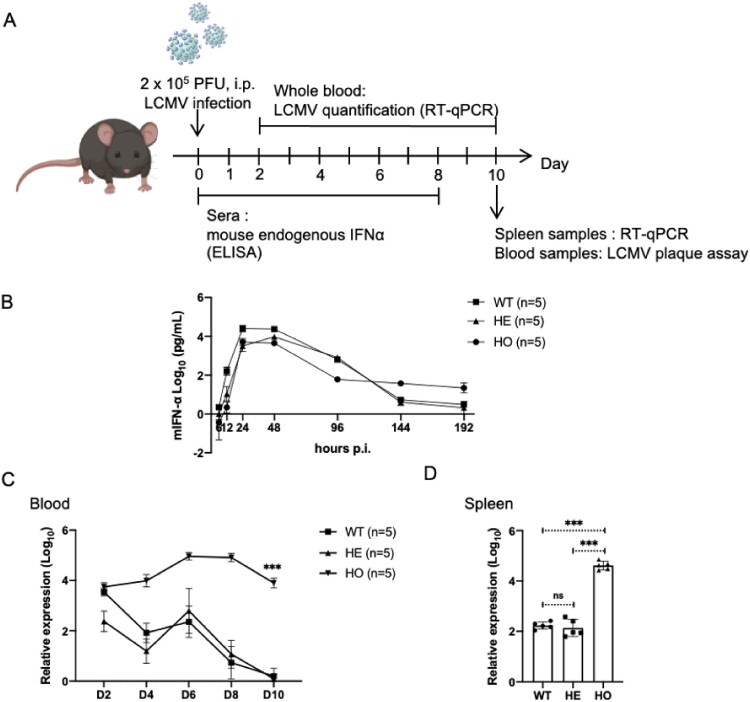
**Heterozygous IFNAR-hEC mice possess intact endogenous IFN-I system**. (A) Schematic model of the study design. (B) Mouse sera were collected at the indicated time points, and the levels of endogenous mIFN-α were quantified using ELISA. (C) Viral load in the blood was assessed by qPCR for NP RNA at different time points post-infection. (D) Viral load in the spleens was evaluated by qPCR for NP RNA on day 10. Statistically significant differences are indicated by * for *p* < 0.05, ** for *p* < 0.01 and *** for *p* < 0.001. Abbreviations: WT, wild-type mouse; HE, heterozygous IFNAR-hEC mouse; HO, homozygous IFNAR-hEC mouse.

To further confirm whether the immune system was impaired by editing murine IFNAR receptor, we conducted a virus challenge experiment using LCMV. We employed wild-type mice (WT), heterozygous IFNAR-hEC mice (HE), and homozygous IFNAR-hEC mice (HO) of C57BL/6N, dividing them into three groups. The mice were intraperitoneally injected with 2 × 10^5^ PFU LCMV-Armstrong (Figure 3A). The induction of mouse endogenous IFN-α was assessed through ELISA and a significant induction of endogenous IFN-α was observed at the early stages of infection, with the peak levels at 24-48 h among the three groups (Figure 3B). RNA extraction was performed on whole blood and spleen samples, followed by LCMV quantification via RT-qPCR using primers targeting the nucleoprotein (NP) protein. An LCMV plaque assay was also performed using blood samples. The results revealed that, in comparison to WT mice and HE mice, HO mice exhibited significantly higher viral titres in blood and spleen samples (Figure 3C-D & Fig. S3A). No significant difference in viral titres was observed between WT mice and HE mice. In addition, we conducted an *ex vivo* experiment using VSV-GFP infected cells derived from ascites fluid (Fig. S3B). The results indicate that peritoneal cells isolated from HO mice exhibited a significantly higher percentage of VSV-GFP positive cells compared to the number observed in both WT mice and HE mice (Fig. S3C). Consequently, the heterozygous IFNAR-hEC mouse retains an intact endogenous IFN-I induction and effector system and can thus be used for studying the antiviral and immunomodulatory functions of human IFN-I.

### Differential activation of JAK-STAT signalling by human IFN-α2 and -α14 subtypes in IFNAR-hEC mice

**Figure 4.**
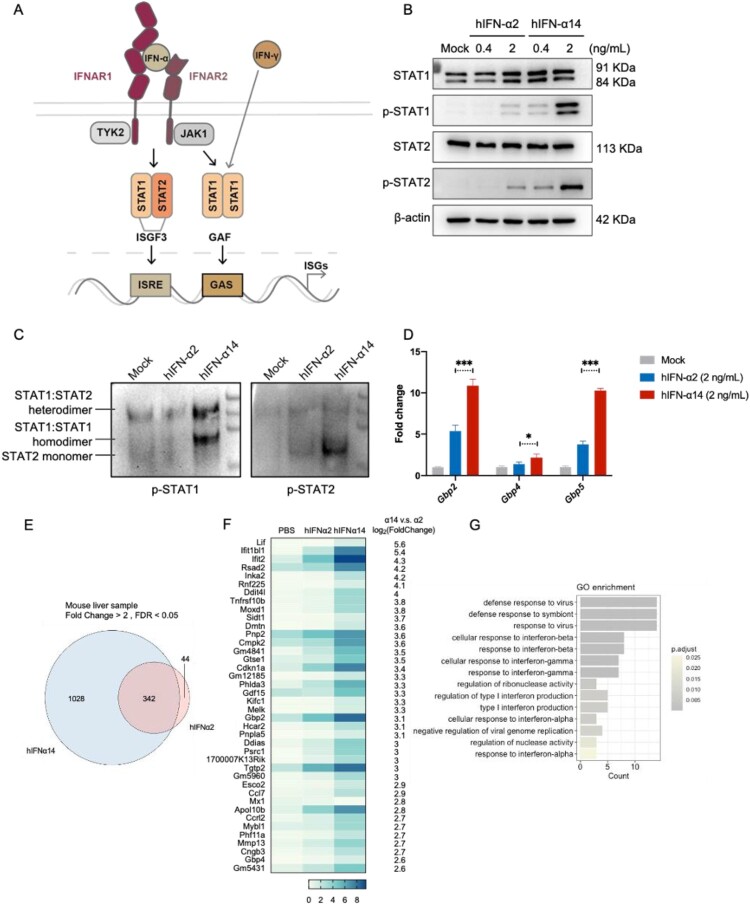
**Effects of human IFN-α2 and -α14 subtypes on IFN-α and -γ signalling in IFNAR-hEC mice**. (A) Schematic model of IFN-I–activated signalling pathways. (B) Primary mouse hepatocytes isolated and treated with human IFN-α2 or IFN-α14 for 30 min were extracted for analysis of p-STAT1(Y701) and p-STAT2(Y690). (C) Primary mouse hepatocytes were treated with 2 ng/mL IFN-α2 or IFN-α14 for 30 min. The STAT1/STAT2 heterodimers and STAT1 homodimers were examined by native PAGE followed by immunoblotting. (D) mRNA levels of GBPs in primary mouse hepatocytes treated with 2 ng/mL hIFN-α for 6 h were determined by RT-qPCR. (E, F) IFNAR-hEC mice were treated with 3.2 μg/kg of IFN-α2 or IFN-α14 every 2 days for 2 weeks. Liver tissue samples were collected six hours after an additional IFN administration. Total RNA was extracted from liver on day 14 for RNA-seq analysis. The heatmap shows differentially expressed genes between IFN-α14-treated and IFN-α2-treated groups. (G) Gene Ontology analysis of differentially expressed genes, showing the top 14 significantly enriched biological processes. Statistically significant differences are indicated by * for *p* < 0.05, ** for *p* < 0.01 and *** for *p* < 0.001.

There are 13 subtypes of IFN-α, with distinct abilities to activate the JAK-STAT pathway and induce antiviral effects in human cells (Figure 4A). IFN-α2 is currently the most widely used interferon in clinical practice, including for the treatment of chronic hepatitis B. According to our previous research and reports from others, among all 13 known human IFN-α subtypes, IFN-α14 has shown very strong antiviral effects against HIV, HBV and SARS-CoV-2 when compared to other subtypes [6, 12, 15]. Therefore, we primarily selected representative IFN-α2 and IFN-α14 as the two subtype models for validation whether IFNAR-hEC mice could be utilized to evaluate distinct effects among human IFN-α subtypes. At equivalent protein concentrations in mouse hepatocytes, IFN-α14 induced higher levels of STAT1 and STAT2 activation, compared with IFN-α2 (Figure 4B). Moreover, compared with IFN-α2 treatment, IFN-α14 exhibited some IFN-γ–like properties that more efficiently induced the formation of STAT1 homodimers, in addition to the classical STAT1-STAT2 heterodimers (Figure 4C & S4A). The phosphorylation of STAT1 and STAT2 was abolished when IFN-I signalling was blocked in the IFN-α14-treated group (Fig. S4B). These findings are highly consistent with our previous observations in human hepatocellular carcinoma cell lines [6]. Similarly, the induction of a specific subfamily of guanosine triphosphatases, known as guanylate binding proteins (GBPs), was significantly greater after IFN-α14 treatment than after IFN-α2 treatment (Figure 4D & S4C). For further validation, we analyzed the transcriptional profiles of IFN-induced genes *in vivo*. The IFN-α14 treated group displayed stronger activation and a distinct ISGs profile, including multiple genes known to be regulated by IFN-γ, compared with the IFN-α2 treated group (Figure 4E-G). We further observed the response to human IFN-α subtypes in the lung, and the results were similar to those observed in liver that IFN-α14 induced a higher level of ISGs compared to IFN-α2 (Fig. S4D). These results suggest that IFNAR-hEC mice can actively respond to human IFN-α14, and support that IFN-α14 can induce a crosstalk between IFN-α and IFN-γ signalling depending on IFNAR [6].

### Human IFN-α14 exhibits robust immunostimulatory activity in IFNAR-hEC mice

**Figure 5.**
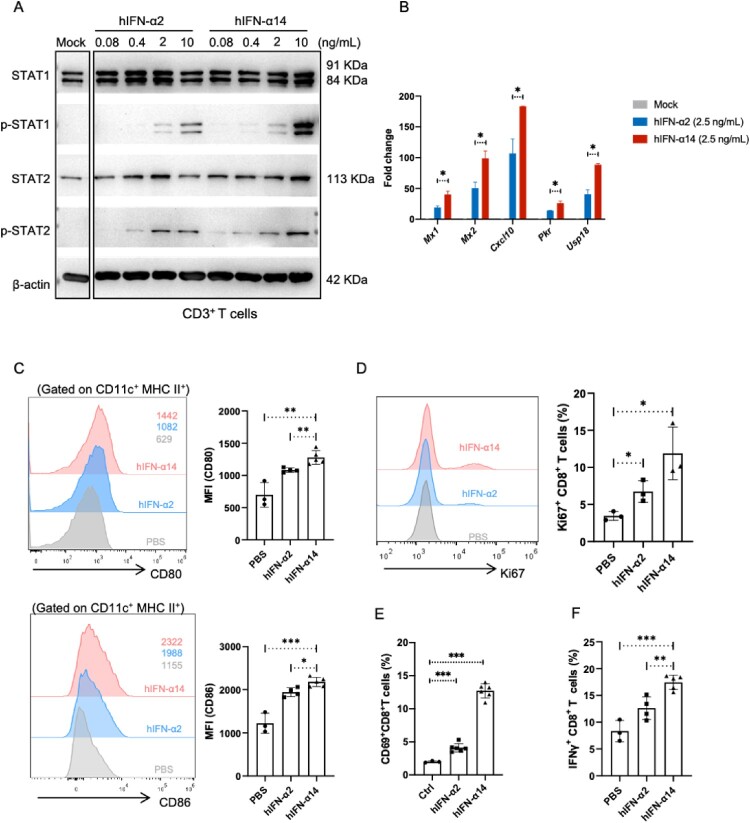
**Effect of human IFN-α2 and IFN-α14 on the activation of dendritic cells and T cells in IFNAR-hEC mice**. (A, B) Mouse CD3^+^ T cells enriched and treated with human IFN-α2 or IFN-α14 for 30 min and 6 h were extracted for analysis of p-STAT1(Y701), p-STAT2(Y690), and expression of ISGs, respectively. (C-F) IFNAR-hEC mice were injected with 3.2 μg/kg of IFN-α2 or IFN-α14; after 6 h, splenocytes were isolated and analyzed by flow cytometry for (C) the expression of CD80 and CD86 on dendritic cells, (E) the percentage of CD69^+^ CD8^+^ T cells, and (F) the percentage of IFN-γ producing CD8^+^ T cells. (D) The percentage of activated Ki67^+^ CD8^+^ T cells was analyzed after treatment with the indicated human IFN-α for 48 h. Statistically significant differences are indicated by * for *p* < 0.05, ** for *p* < 0.01 and *** for *p* < 0.001.

Because IFNAR-hEC mice were immunocompetent, we further investigated the effects of IFN-α2 and -α14 on adaptive immune cells. When CD3^+^ T cells were isolated and treated with equal quantities of IFN-α2 and IFN-α14, we found that IFN-α14 induced greater phosphorylation of STAT1 and STAT2 (Figure 5A, Fig. S5A), and higher levels of ISGs (Figure 5B, Fig. S5B-C), similar to the findings in PMHs. When IFNAR-hEC mice were injected with 3.2 μg/kg of IFN-α2 or IFN-α14, we observed that IFN-α14 treatment led to greater upregulation of the expression levels of CD80 and CD86 co-stimulatory molecules on dendritic cells (DCs), compared with IFN-α2 (Figure 5C). Additionally, IFN-α14 treatment led to greater upregulation of the expression levels of CD69 and Ki67 on CD8^+^ T cells (Figure 5D-E), and resulted in higher frequencies of IFN-γ-producing CD8^+^ T cells (Figure 5F), compared with IFN-α2 treatment. We tested the activation of lymphocytes isolated from the lung, and the results showed that IFN-α14 exhibited an enhanced immunomodulatory activity, as indicated by higher CD69 levels and greater cytokine secretion capacity of CD8^+^ T cells (Fig. S5D-E).

**Figure 6.**
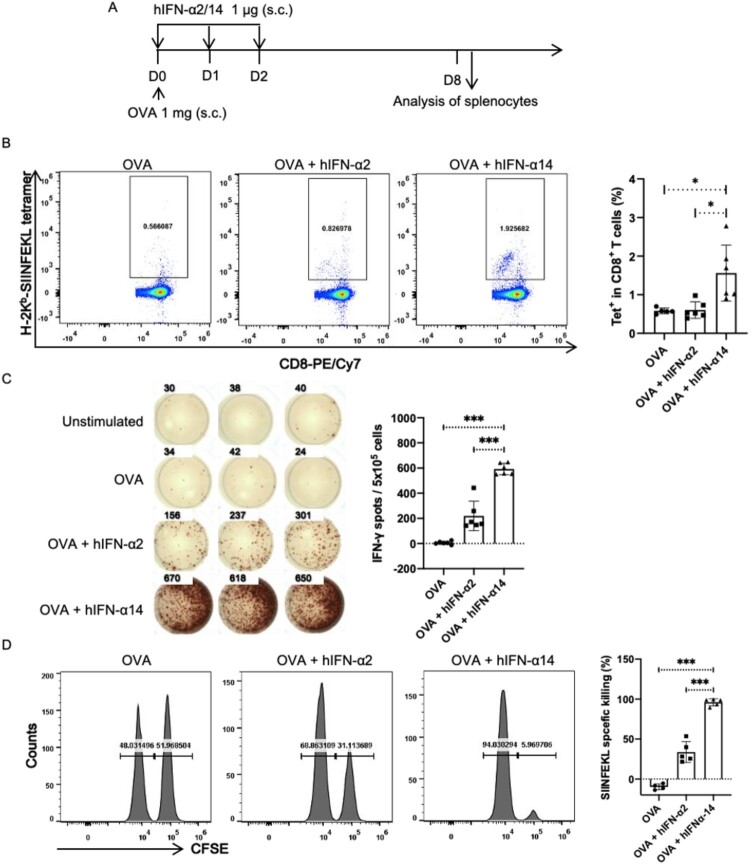
**IFN-α14 more effectively promotes cross-priming of CD8^+^ T cells**. (A) IFNAR-hEC mice were immunized by subcutaneous injection with OVA alone or with OVA and human IFN-α; 8 days later, splenocytes were isolated and analyzed for SIINFEKL-specific CD8^+^ T cells. (B, C) The percentage of SIINFEKL-specific CD8^+^ T cells was quantified by MHC I tetramer staining and IFN-γ ELISPOT assays. (D) Lymphocytes from wild-type mice were pulsed with OVA_257-264_ peptide and labelled with CFSE as target cells, then transferred into recipient mice that had been immunized for 7 days. After 24 h, splenocytes from recipient mice were isolated to determine cytotoxic activity in SIINFEKL-specific CTLs. Statistically significant differences are indicated by * for *p* < 0.05, ** for *p* < 0.01 and *** for *p* < 0.001.

Furthermore, we investigated the effects of IFN-α2 and -α14 on antigen presentation and cross-priming process in CD8^+^ T cells using an established protocol that involved the soluble protein ovalbumin (OVA) [16] (Figure 6A). After injection with OVA alone, we observed few SIINFEKL-specific CD8^+^ T cells via major histocompatibility complex class I (MHC-I) tetramer staining or IFN-γ ELISPOT assay. In contrast, injection with OVA and IFN-α2 slightly increased the proportion of SIINFEKL-specific CD8^+^ T cells and production of IFN-γ; injection with OVA and IFN-α14 led to more robust responses (Figure 6B-C). To confirm that IFN-α14 promoted cross-priming in CD8^+^ T cells, we explored whether the immunization-generated SIINFEKL-specific cells were functional by measuring cytotoxic T lymphocyte (CTL) activity. Similar to the antigen presentation findings, we observed few SIINFEKL peptide-pulsed cells were killed by splenocytes from mice immunized with OVA alone, whereas splenocytes from mice injected with OVA and IFN-α displayed substantial cytotoxicity against the target cells; injection with IFN-α14 led to a more pronounced effect than injection with IFN-α2 (Figure 6D). In summary, these results indicate that the established IFNAR-hEC mouse model can be used to investigate distinct immunostimulatory activities among IFN-I subtypes; moreover, compared with IFN-α2, human IFN-α14 exhibits greater immunostimulatory activity in IFNAR-hEC mice.

### Viral suppression in IFNAR-hEC mice with HBV replication upon treatment with clinically-used PEGylated hIFN-α2

**Figure 7.**
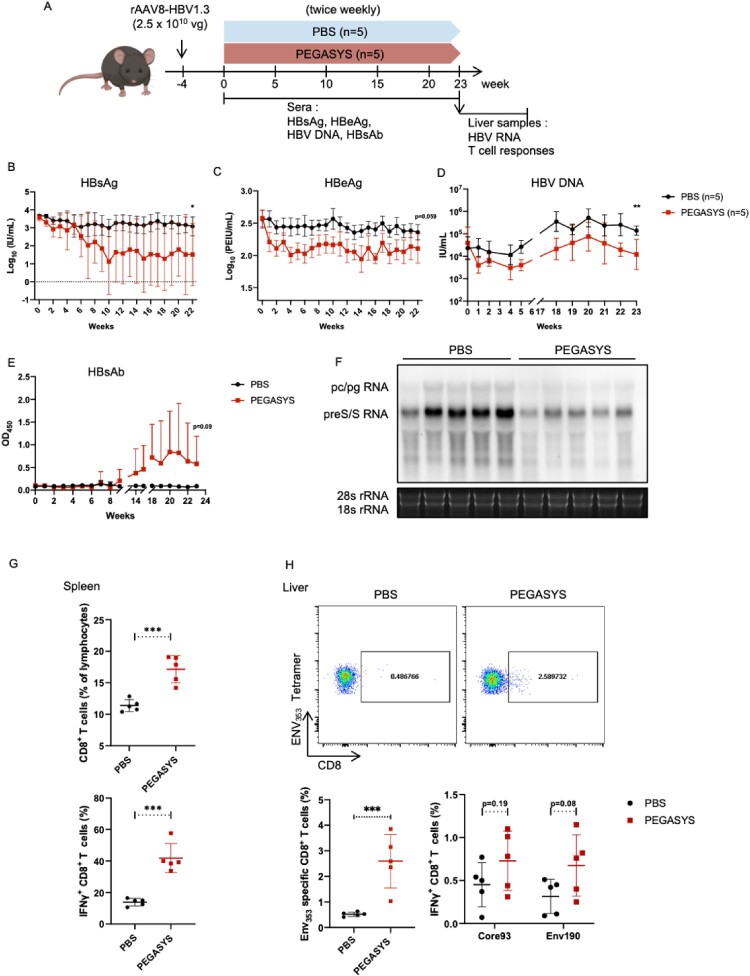
**Antiviral and immunomodulatory activities of PEGylated IFN-α2 in IFNAR-hEC mice with HBV replication**. An AAV/HBV mouse model was established by intravenous administration of 2.5 × 10^10^ copies of recombinant adeno-associated virus serotype 8 carrying the HBV genome (rAAV8-HBV1.3). Mice were grouped according to day 0 serum HBsAg level, then subcutaneously injected with 30 μg/kg PEGASYS or an equal volume of phosphate-buffered saline (PBS), as indicated (A). Blood was collected weekly and tested for (B) HBsAg, (C) HBeAg, (D) HBV DNA and (E) HBsAb. Mice were sacrificed at the administration endpoint; total RNA was extracted from liver samples for detection of HBV RNA via Northern blotting (F). Splenocytes were isolated and analyzed for the frequency and production of IFN-γ of total CD8^+^ T cells (G). An ENV_353_-specific subset of lymphocytes in liver was labelled by the anti-CD8 antibody and ENV_353_ tetramer, and was analyzed by flow cytometry. The responsiveness of specific intrahepatic T cell to Core_93_ or Env_190_ were tested with an intracellular staining assay for IFN-γ (H). Statistically significant differences are indicated by * for *p* < 0.05, ** for *p* < 0.01 and *** for *p* < 0.001.

Because many receptors are species-specific, few animal models can be used to assess the immunomodulatory functions of human IFN-α and its long-acting versions used in clinical practice. In this study, we explored the activation of virus-specific adaptive immunity in IFNAR-hEC mice using pegylated IFN-α2a (PEGASYS, Roche) as a representative drug. An adeno-associated virus (AAV)/HBV carrier mouse model with long-term HBV persistence was established to mimic the immune tolerance status in patients with HBV infection. Mice were grouped according to day 0 serum HBsAg level; they received a subcutaneous injection of 30 μg/kg PEGASYS [17] or equal volume of PBS (Figure 7A). Blood was collected weekly and tested for HBsAg (Figure 7B), HBeAg (Figure 7C), HBV DNA (Figure 7D) and HBsAb (Figure 7E, Fig. S6A). Mice were sacrificed and total RNA from liver samples was used to detect HBV RNA (Figure 7F). In the PEGASYS-treated group, mice exhibited a rapid decrease in the HBV antigen level, which remained low and was accompanied by the production of HBsAb after 9 weeks. Additionally, PEGASYS treatment effectively reduced HBV DNA and RNA levels accompanied by an elevation in both ALT and AST (Fig. S6B). We found that PEGASYS treatment led to activation of the overall T-cell response (Figure 7G). Examination of HBsAg-specific T-cell responses at the end point revealed that the percentage of Env_353_-specific T cells in liver was significantly increased (Figure 7H). Moreover, both HBsAg-specific and HBcAg-specific CD8^+^ T cells displayed enhanced cytokine secretion capacities for IFN-γ (Figure 7H). Besides, when mice were sacrificed after treatment of PEGASYS for 4 weeks, detectable and augmented activation of total and HBV-specific CD8^+^ T cell responses were observed (Fig. S6C). Collectively, these findings indicate that IFNAR-hEC mice display robust and sustained responsiveness to PEGASYS, resulting in HBV suppression and adaptive immune activation. IFNAR-hEC mice can be utilized to evaluate the antiviral immunomodulatory effects of human IFN-α treatments used in clinical practice.

## Discussion

Mice are commonly used as animal models in IFN-I studies [18, 19]. However, because of their limited similarity (less than 50%) with human IFNAR and the species-specific nature of IFN-I, mice are not the best model for these investigations [11, 20, 21]. Furthermore, in models such as human liver chimeric mice [17] and patient-derived xenograft (PDX) models [22, 23], only specific tissues or organs containing human cells are capable of responding to human IFNs. The evaluation of immune modulation remains limited because of the inherent immune deficiencies of these models. Although humanized immune system mouse models [24] theoretically can evaluate differential efficacy among immune cells, their high cost, long model generation period and the limited chimerism rate hinder widespread application. In the present study, we established a novel IFNAR-hEC mouse model, allowing robust responses to human IFN-I throughout the body. Importantly, this model exhibits distinct effect of human IFN-α subtypes, while reflecting the comprehensive immune responses induced by human IFN-I because of its competent immune system.

IFNAR-hEC mice provide a good model for assessments of IFN-I-based drugs, including drugs derived from IFN-α, IFN-β, and ΙFN-ω. A hybrid IFNAR mouse model with a human extracellular domain was previously reported; however, its strategy involved constitutive expression of hybrid IFNAR using the mouse PGK1 promoter, which may result in similar levels of protein expression across various tissues. Additionally, it was unclear whether this model can reveal the distinct effects of IFN-α subtypes [11]. Here, we chose to retain the regulated and signal sequences of mouse *Ifnar1/2* to faithfully reproduce the physiological tissue distribution pattern of the corresponding receptors. Additionally, we confirmed the distinct activation patterns of the JAK-STAT pathway induced by IFN-α2 and IFN-α14 subtypes in immunocompetent heterozygous IFNAR-hEC mice, both at the *ex vivo* and *in vivo* levels. Compared to IFN-α2, exposure to an equivalent dose of IFN-α14 demonstrated a synergistic activation effect on both IFN-α and -γ signalling pathways, consistently with our findings in human cells [6]. This unique synergistic activation effect of IFN-α14 could serve as the scientific basis for its better immunomodulatory function.

In clinical settings, pegylated IFN-α2 can reduce HBV DNA and antigens to varying degrees, but its efficacy in achieving sustained suppression of HBV is limited (with an overall 3%∼10% HBsAg seroconversion rate in HBeAg-positive individuals), often accompanied by a series of side effects [8]. We tested the antiviral effect of the clinically used PEG-IFN-α2a in the IFNAR-hEC mice-based AAV/HBV model. Suppression of HBV DNA, RNA, and antigen expression was observed, and two of the five mice receiving PEG-IFN-α2a showed a certain induction of HBsAb. Of note, although prolonged treatment could result in a higher seroconversion rate of HBsAg in more mice, the efficacy of IFN-α2 remains limited. Therefore, ongoing research focuses on exploring novel IFN subtypes and formulations, and the present model provides a robust platform to study the efficacy and antiviral mechanisms of various human IFN-I subtypes *in vivo*. Recombinant proteins of various subtypes, such as IFN-α14, have shown promising antiviral effects among the 13 known IFN-α subtypes [15, 25]. Furthermore, fusion proteins that combine IFNs with other molecules, including PD-1 antibodies for enhanced immunomodulatory effects [26] or ASGPR for enhanced targeting [18], are in development, along with long-acting IFN strategies such as PASylated IFN [27]. Additionally, novel IFN formulations and delivery systems are under investigation to improve drug delivery and efficacy [28]. Therefore, humanized IFNAR mice offer a valuable tool for the evaluation of emerging IFN-based therapies, including treatments for viral hepatitis, respiratory illnesses (e.g. COVID-19), HCC and other tumours. The model also can be used to assess the synergistic effects and potential benefits of combining IFN-Is with drugs such as siRNA or antiviral antibodies, which will allow researchers to explore novel treatment regimens and optimize combination strategies for enhanced antiviral or immunomodulatory effects. Another important aspect of IFN research involves the analysis of side effects. Previous studies primarily relied on wild-type mice when assessing the toxicity of IFN-α used in clinical practice [29]. However, considering the species specificity of IFN-I/IFNAR, it is difficult to accurately reflect the side effect profile in wild-type mice. The development of the IFNAR-hEC mouse model addresses the limitations of using wild-type mice for assessments of IFN toxicity; it will enable researchers to more accurately evaluate the potential side effects of human IFNs. Overall, this model will be a valuable tool for assessing the effectiveness and tolerability of IFN-based therapeutics, facilitating the development of more potent and targeted IFN therapies.

Although humanized IFNAR mice have significant advantages in terms of evaluating human IFN-I and its subtypes, it is important to acknowledge limitations regarding the potential impacts of receptor subunit pairing and differences in downstream signalling. In the present study, heterozygous mice were used to ensure that the mice responded to human IFNs and maintained responsiveness to endogenous murine IFNs. However, the potential effects of mouse receptor subunit and human receptor subunit pairing in the formation of IFNAR could not be completely ruled out. Nonetheless, because of species differences and the low level of IFNAR sequence conservation between human and mice, we presume that the impact of such pairing on the model is minimal. Furthermore, in this model, the extracellular domain of the chimeric receptor was humanized, whereas the transmembrane and intracellular domains of IFNAR retained their murine structure. These differences from fully human IFNAR may affect the functional efficacies of various human IFN subtypes [30]. Moreover, although IFNAR receptors are humanized, the molecules involved in downstream intracellular signalling and ISGs are still mouse-derived. However, considering the relative conservation of the downstream IFN-I pathway and our previous findings [6], as well as the findings regarding signalling activation and functionality presented in this study, IFNAR-hEC mice appear to effectively reflect the effects of different IFN-I subtypes. The factors mentioned above should be considered when designing future studies and analyzing related results using this model to gain a more accurate understanding of the *in vivo* effects of human IFN subtypes.

Altogether, the generation of extracellular-humanized IFNAR1/2 mice, displaying an active response to human IFN-I, has facilitated the investigation into the distinct activities of human IFN-I subtypes, particularly their differential effects on both the innate immune signalling pathway and immunomodulatory functions. This will contribute to advancing our understanding of IFN biological functions and promoting the development of improved IFN-based therapeutic strategies.

## Supplementary Material

Supplemental Material
