# Supplementary material for: An extracellular humanized IFNAR immunocompetent mouse model for analyses of human interferon alpha and subtypes
Source: Emerg Microbes Infect. 2023 Nov 23;13(1):2287681. doi: 10.1080/22221751.2023.2287681 (PMC10810641; doi:10.1080/22221751.2023.2287681)
Supplement: Supplemental Material [file TEMI_A_2287681_SM5967.docx]

**Supplementary Materials**

**Materials and Methods**

***Cell line, Mice and Viral infections***

The HepG2-NTCP cell line was provided by Prof. Stephan Urban. Wild type mice, heterozygous IFNAR-hEC mice and homozygous IFNAR-hEC mice of C57BL/6N strain were aged 6-8 weeks and bred in the same room. Mice were intraperitoneally injected with 2 x 10^5^ PFU of LCMV-Armstrong. An AAV/HBV mouse model was established by intravenous administration of 2.5 x 10^10^ copies of recombinant adeno-associated virus serotype 8 carrying the HBV genome (rAAV8-HBV1.3).

***RNA extraction and Real-Time Quantitative PCR (RT-qPCR) analysis***

Total RNA was extracted using TRIzol reagent. The mRNA levels of IFN-α receptors, IFN-stimulated genes (*GBP2/4/5*, *ISG15*, *PKR, USP18, Mx1/2 and CXCL10*) and HBV RNAs were quantified with real-time PCR using SYBR green master mix. The primers used are listed in Table S2.

***Western blot and Northern blot***

Western blot and Northern blot were conducted as previously reported [1,2]. Native polyacrylamide gel electrophoresis was used to detect homodimers and heterodimers as we previously described [3].

***Lymphocyte preparation and flow cytometry analysis***

Mice were perfused through the vena cava with PBS, then subjected to liver and spleen extraction. After the liver had been digested with type IV collagenase for 30 minutes, it was minced and dissociated using a 70-µm cell strainer. Next, the cell pellet was resuspended with 40% Percoll solution and red blood cells were removed using RBC lysis buffer. Finally, intrahepatic lymphocytes were washed and resuspended in FACS buffer or RPMI 1640 medium. Splenic lymphocytes and PBMCs were directly isolated by removal of red blood cells. MojoSort mouse CD3 selection kit (480100, Biolegend) was used for mouse CD3^+^ T cells isolation.

For isolation lymphocytes from lung, the minced tissue was subjected to digestion in a solution containing 1 mg/mL of type II collagenase (C6885, Sigma) diluted with RPMI 1640 medium. The mixture was incubated at 37°C for 1 hour and subsequently passed through a 70 μm cell strainer. The cell pellet was centrifuged at 450 g, room temperature, for 5 minutes. Red blood cells were removed using RBC lysis buffer. Finally, the isolated lymphocytes were washed and resuspended in either FACS buffer or RPMI 1640 medium.

For isolation lymphocytes from peritoneal fluid, a 5 mL syringe containing 4 mL PBS was gently injected into the abdominal cavity, followed by careful agitation of the mouse. Subsequently, peritoneal lavage fluid was aspirated from the peritoneal side using a 5 mL syringe. The collected solution was then subjected to centrifugation for 10 minutes at 1000 rpm, and the resulting cell pellet was washed and resuspended in RPMI 1640 medium.

For cell surface marker staining, lymphocytes were suspended in FACS buffer and labeled with the corresponding antibodies at 4°C for 30 minutes. For MHC-I peptide tetramer staining, isolated cells were incubated for 10 minutes at room temperature with MHC-I peptide tetramers conjugated with H-2Kb/VWLSVIWM for HBsAg (Env353; HelixGen, China) or H-2Kb/SIINFEKL for OVA (OVA257; HelixGen, China), then stained with surface antibodies. For intracellular cytokine staining, lymphocytes were plated on 96-well plates (1 × 106 cells per well), then incubated for 6 hours with 5 µg/ml of HBsAg peptide (epitope Env190-197; ChinaPeptides Co., Ltd.) or HBcAg peptide (epitope Core93-100; ChinaPeptides Co., Ltd.), in the presence of 1 µg/ml brefeldin A (B5936, Sigma-Aldrich). After stimulation, cells were fixed and permeabilized for 20 minutes, then stained for surface markers, and finally stained with antibodies against intracellular cytokines. Samples were analyzed using an Attune NxT flow cytometer (Thermo Fisher), and data were analyzed with FlowJo software.

***LCMV-Plaque Assay***

Vero cells were plated on 6-well plates, and the mouse serum was diluted with DMEM culture medium and the cells were infected with the diluted solution for 1 hour. Next, add 3 mL of overlay (consists of 2 x MEM medium and 2% agarose gel in a ratio of 1:1) to each well and incubate at 37℃, 5% CO_2_ for another 4 days. Finally, the cells were fixed in 7% formaldehyde for 1 hour and stained with crystal violet for 30 minutes. After the plates were washed with water and left out to dry, we could observe the formation of plaques.

***RNA sequencing analysis***

RNA samples were prepared using TRIzol reagent. RNA sequencing was conducted by GENEWIZ Inc. (Suzhou, China). mRNA libraries were prepared with the VAHTS mRNA-seq V3 Library Prep Kit for Illumina (Vazyme, NR611), in accordance with the manufacturer’s instructions, then sequenced on an Illumina NovaSeq 6000 instrument. Data in FASTQ format were filtered using Cutadapt (V1.9.1) to eliminate sequences with base quality < 20. Hisat2 (V2.0.1) was used to index reference genome sequences and align cleaned data. Aligned reads were filtered and sorted using SAMtools (V1.8). The DESeq2 package (V1.34.0) was used for differential expression analysis. The VennDiagram package (1.7.3) was used to generate Venn diagrams of differential genes. The ClusterProfiler package (4.2.2) was used to identify Gene Ontology terms enriched in biological processes. Heatmaps were generated with GraphPad Prism 8.0 (GraphPad Software Inc.).

***Serum analysis***

The mouse serum HBsAg and HBeAg concentrations were quantified using the HBsAg detection ELISA kit (Antubio, China), and were assayed for HBsAb qualitatively using ELISA plates coated with CHO-HBsAg. HBV DNA was determined by qPCR using Hepatitis B viral DNA Quantitative Fluorescence Diagnostic Kit (Shengxiang, Hunan, China). The serum levels of AST and ALT were detected by Servicebio Technology (Wuhan) Co. Mouse endogenous IFN-α was assessed using the Mouse IFN-Alpha All Subtype ELISA Kit (42115-1, PBL Assay Science).

**Table S1. Antibodies used in this study.**

| **Antibodies** | **Cat. NO.** | **Origin** |
| --- | --- | --- |
| Anti-Stat1 Ab | 14994S | CST |
| Anti- phospho-Stat1 (Tyr701) Ab | 9167S | CST |
| Anti-Stat2 Ab | 72604S | CST |
| Anti-phospho-Stat2 (Tyr689) Ab | 07-224 | Millipore |
| Anti-β-actin Ab | A2522 | Sigma-Aldrich |
| Anti-IFNAR1-PE(MMHAR-3) Ab | 21370-3 | PBL |
| Anti-IFNAR2-FITC Ab | 10359-R122-F | Sino Biological |
| Anti-CD3-FITC Ab | 100204 | Biolegend |
| Anti-CD4-Pacific Blue Ab | 116008 | Biolegend |
| Anti-CD8-PE/Cy7 Ab | 100722 | Biolegend |
| Anti-NK1.1-BV605 Ab | 108740 | Biolegend |
| Anti-B220-FITC Ab | 103206 | Biolegend |
| Anti-CD69-APC Ab | 104514 | Biolegend |
| Anti-Ki67-PE Ab | 12-5698-82 | eBioscience |
| Anti-CD11c-FITC Ab | 117305 | Biolegend |
| Anti-MHC II-PE Ab | 107607 | Biolegend |
| Anti-CD80-APC Ab | 104714 | Biolegend |
| Anti-CD86- Alexa Fluor700 Ab | 105024 | Biolegend |
| Anti-IFN-γ-BV711 Ab | 505836 | Biolegend |
| Anti-TNF-α-PE Ab | 506306 | Biolegend |
| Anti-Human IFNAR2 nAb | 21385-1 | PBL |
| Anti-GBP2 Ab | 11854-1-AP | Proteintech |
| Anti-PKR Ab | R22791 | Zenbio |

**Table S2. Primers used in this study.**

| **Primer** | **Sequences (5’-3’)** |
| --- | --- |
| IFNAR1-F | CCGTACTGGTCATTACTGTGGTT |
| IFNAR1-R1 | ACCAAATGCTTCCCACATTAAAAGGA |
| IFNAR1-R2 | CACTGAACTTGAAAGGTCATGTTTGC |
| IFNAR2-F | CCACATTACCCAAGAGCATCCATAC |
| IFNAR2-R1 | CCTCTACCTAGAAAGGATTCCAATAAACTG |
| IFNAR2-R2 | ATTGTGTGAGCAACTGAACAACGT |
| mIFNAR1-mRNA-s﻿ | AGAATATCGAACAAAAGACGAGGCGAA |
| mIFNAR1-mRNA-as | CTGTGTTTTGATATACACATTTGTGTCCAG |
| mIFNAR2-mRNA-s | GTCATAGTGCACAGAGGGGACTTG |
| mIFNAR2-mRNA-as | TGTGGTCTGTAAAGCCAACGATCTCA |
| IFNAR1-hEC-mRNA-s | GAGGTGGAACAGGAGCGATGAG |
| IFNAR1-hEC-mRNA-as | ﻿CCCAGACAATTTTATCCAATTATCCATCCC |
| IFNAR2-hEC-mRNA-s | GCGAAATTTCCGGTCCATCTTATCATG |
| IFNAR2-hEC-mRNA-as | CCACCTTCAAATCTTCTGGTTTACTCAT |
| mGAPDH-s | TGACCTCAACTACATGGTCTACA |
| mGAPDH-as | CTTCCCATTCTCGGCCTTG |
| mMx1-s | GACCATAGGGGTCTTGACCAA |
| mMx1-as | AGACTTGCTCTTTCTGAAAAGCC |
| mMx2-s | GAGGCTCTTCAGAATGAGCAAA |
| mMx2-as | CTCTGCGGTCAGTCTCTCT |
| mPKR-s | CTGGTTCAGGTGTCACCAAAC |
| mPKR-as | ACAACGCTAGAGGATGTTCCG |
| mISG15-s | GATTGCCCAGAAGATTGGTG |
| mISG15-as | TCTGCGTCAGAAAGACCTCA |
| mUSP18-s | CCAAACCTTGACCATTCACC |
| mUSP18-as | ATGACCAAAGTCAGCCATCC |
| mCXCL10-s | GGGCCATAGGGAAGCTTGAAA |
| mCXCL10-as | TCATCGTGGCAATGATCTCAAC |
| mGBP2-s | CTTGACACTGAGGGCCTTGAA |
| mGBP2-as | TGCCAAAGCAAAGATCCAGC |
| mGBP4-s | ACTACTGGCAGGTTCCCAGG |
| mGBP4-as | GCTTCTCTGCAATGGCCTTCT |
| mGBP5-s | CTTTATCGCACAGGCAAATCCTACC |
| mGBP5-as | CCCTTGGTGTGAGACTGTACAGT |
| mβ-actin-s | CCTGGTGCCTAGGGCG |
| mβ-actin-as | CGCGTCCACCCGCGAG |
| hHIF-1α-s | GAACGTCGAAAAGAAAAGTCTCG |
| hHIF-1α-as | CCTTATCAAGATGCGAACTCACA |
| mHIF-1α-s | GGGGAGGACGATGAACATCAA |
| mHIF-1α-as | GGGTGGTTTCTTGTACCCACA |
| hIRF1-s | CTGTGCGAGTGTACCGGATG |
| hIRF1-as | ATCCCCACATGACTTCCTCTT |
| mIRF1-s | GGCCGATACAAAGCAGGAGAA |
| mIRF1-as | GGAGTTCATGGCACAACGGA |
| hMx1-s | GGTGGTCCCCAGTAATGTGG |
| hMx1-as | CGTCAAGATTCCGATGGTCCT |
| hOAS1-s | AAGGCTGGAATTTCATTCTC |
| hOAS1-as | CATTTTCAGGTGGGACTCT |
| mOAS1-s | ﻿AGGTCCAGAGTTCATGGTGGC |
| mOAS1-as | CAGGCTTGCTGGAAGTATTAACATGAC |
| NP-s | CAGAAATGTTGATGCTGGACTGC |
| NP-as | CAGACCTTGGCTTGCTTTACACAG |

**Table S3. Commercial interferon used in this study.**

| **Interferon** | **Cat. NO.** | **Origin** |
| --- | --- | --- |
| hIFN-α1 | 11125-1 | PBL |
| hIFN-α2a | 11100-1 | PBL |
| hIFN-α4 | 11180-1 | PBL |
| hIFN-α5 | 11135-1 | PBL |
| hIFN-α6 | 11165-1 | PBL |
| hIFN-α7 | 11160-1 | PBL |
| hIFN-α8 | 11115-1 | PBL |
| hIFN-α10 | 11120-1 | PBL |
| hIFN-α14 | 11145-1 | PBL |
| hIFN-α16 | 11190-1 | PBL |
| hIFN-α17 | 11150-1 | PBL |
| hIFN-α21 | 11130-1 | PBL |
| hIFN-β | 11420-1 | PBL |
| hIFN-ω | 11395-1 | PBL |
| hIFN-γ | 300-02 | PeproTech |
| mIFN-α1 | 12105-1 | PBL |

**Reference**

1. Zhang W, Chen J, Wu M, et al. PRMT5 restricts hepatitis B virus replication through epigenetic repression of covalently closed circular DNA transcription and interference with pregenomic RNA encapsidation. Hepatology. 2017 Aug;66(2):398-415.

2. Shen F, Li Y, Wang Y, et al. Hepatitis B virus sensitivity to interferon-alpha in hepatocytes is more associated with cellular interferon response than with viral genotype. Hepatology. 2018 Apr;67(4):1237-1252.

3. Chen J, Wu M, Zhang X, et al. Hepatitis B virus polymerase impairs interferon-α-induced STA T activation through inhibition of importin-α5 and protein kinase C-δ. Hepatology. 2013 Feb;57(2):470-82.

**
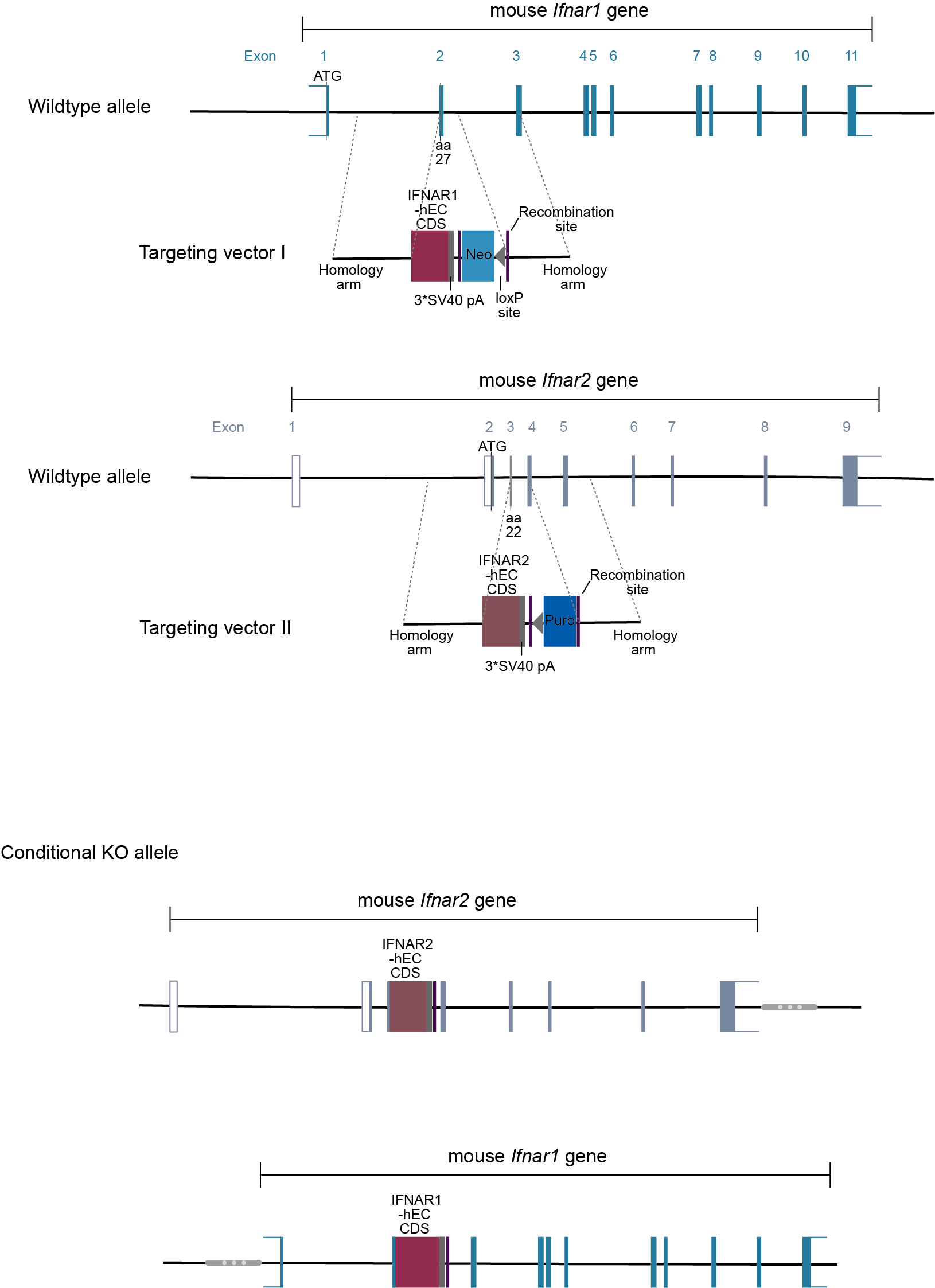
**

**Figure S1. Schematic representation of the knock-in strategy for IFNAR-hEC coding genes.** The mouse *Ifnar1* and *Ifnar2* genes are both located on mouse chromosome 16. The first targeting region spanned from the ATG start codon (aa.27) in exon 2 through a portion of intron 2 in mouse *Ifnar1*; the second targeting region spanned from the ATG start codon (aa.22) in exon 3 through a portion of intron 3 in mouse *Ifnar2*. A loxP site was used to determine whether the two targeting regions were on the same chromosome. Each targeting vector contained the humanized receptor coding fragment and a resistance gene; the resistance gene was excised via recombination after mating.

**
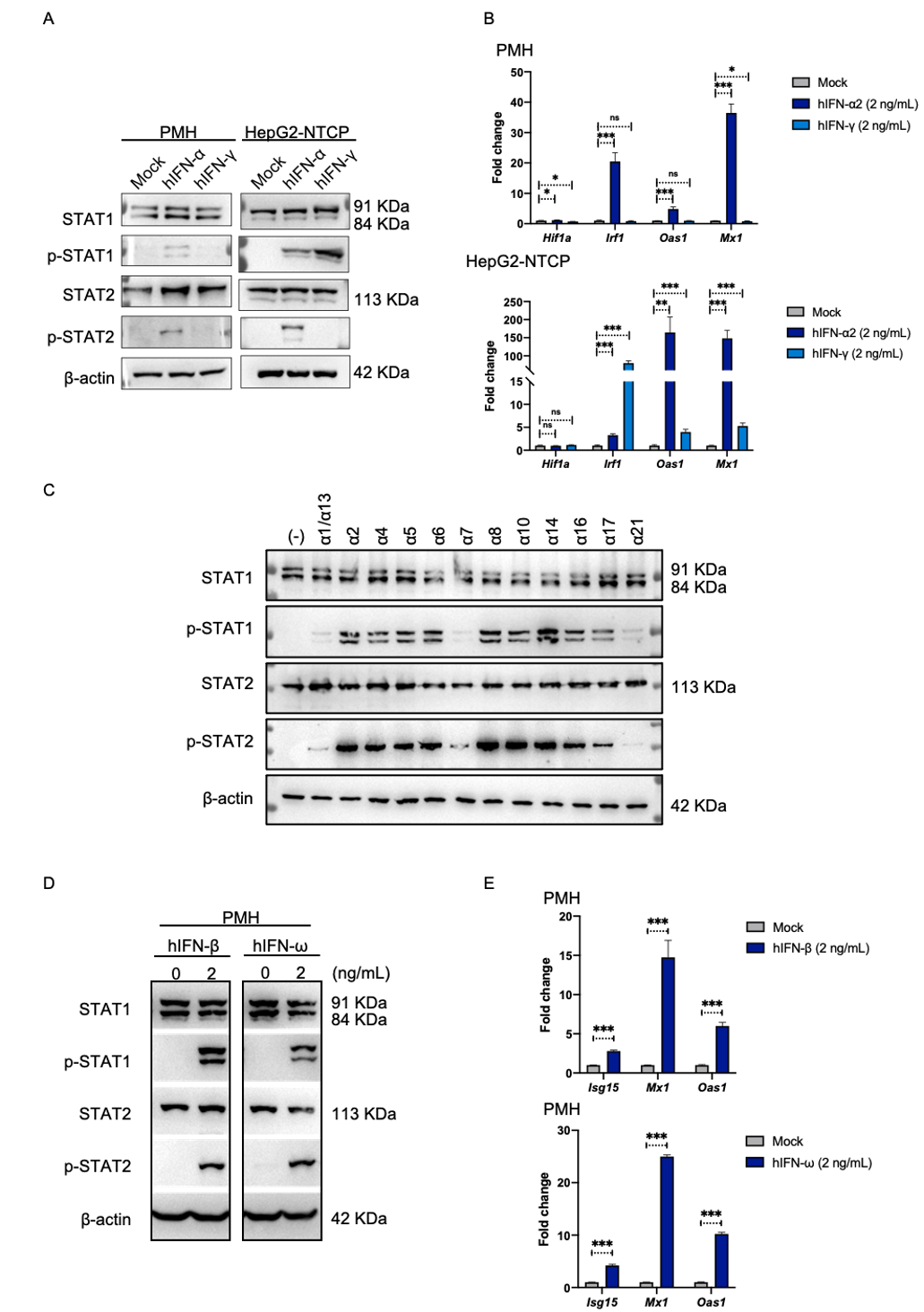
 Figure S2. Responses of IFNAR-hEC mice to treatment with human IFN-Is.** (A, B) Primary mouse hepatocytes treated with 2 ng/mL human IFN-α2, human IFN-γ for 30 minutes and 6 hours were extracted for analysis of (A) p-STAT1(Y701), p-STAT2(Y690), and expression of (B) ISGs, respectively. HepG2-NTCP were treated with human IFN-α2 or IFN-γ as positive controls. (C) Primary mouse hepatocytes were treated with 10 ng/mL human IFN-α subtypes for 30 minutes, and were extracted for analysis of p-STAT1(Y701), p-STAT2(Y690). (D, E) Primary mouse hepatocytes treated with 2 ng/mL human IFN-β, human IFN-ω for 30 minutes and 6 hours were extracted for analysis of (D) p-STAT1(Y701), p-STAT2(Y690), and expression of (E) ISGs, respectively. Statistically significant differences are indicated by * for p < 0.05, ** for p < 0.01 and *** for p < 0.001. Abbreviations: PMH, primary mouse hepatocytes; NTCP, sodium taurocholate co-transporting polypeptide.


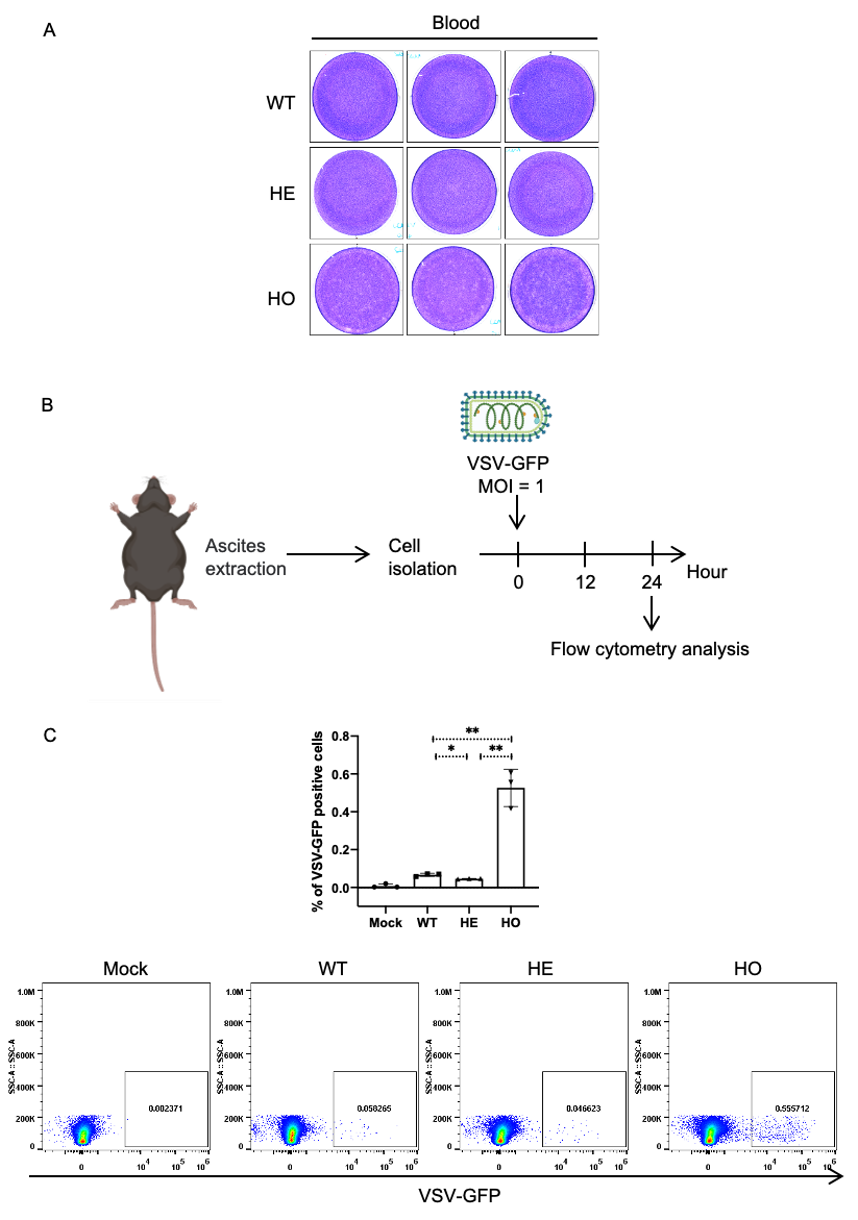


**Figure S3. Heterozygous IFNAR-hEC mice retain an intact endogenous IFN-I system. (A) Viral load of LCMV in the blood was assessed through plaque assay on day 10.** (B) Schematic model of the study design. (C) The percentage of virus-infected cells (GFP-positive cells) was quantified by flow cytometry. Statistically significant differences are indicated by * for p < 0.05 and ** for p < 0.01. Abbreviations: WT, wild-type mouse; HE, heterozygous IFNAR-hEC mouse; HO, homozygous IFNAR-hEC mouse. MOI, multiplicity of infection.

**
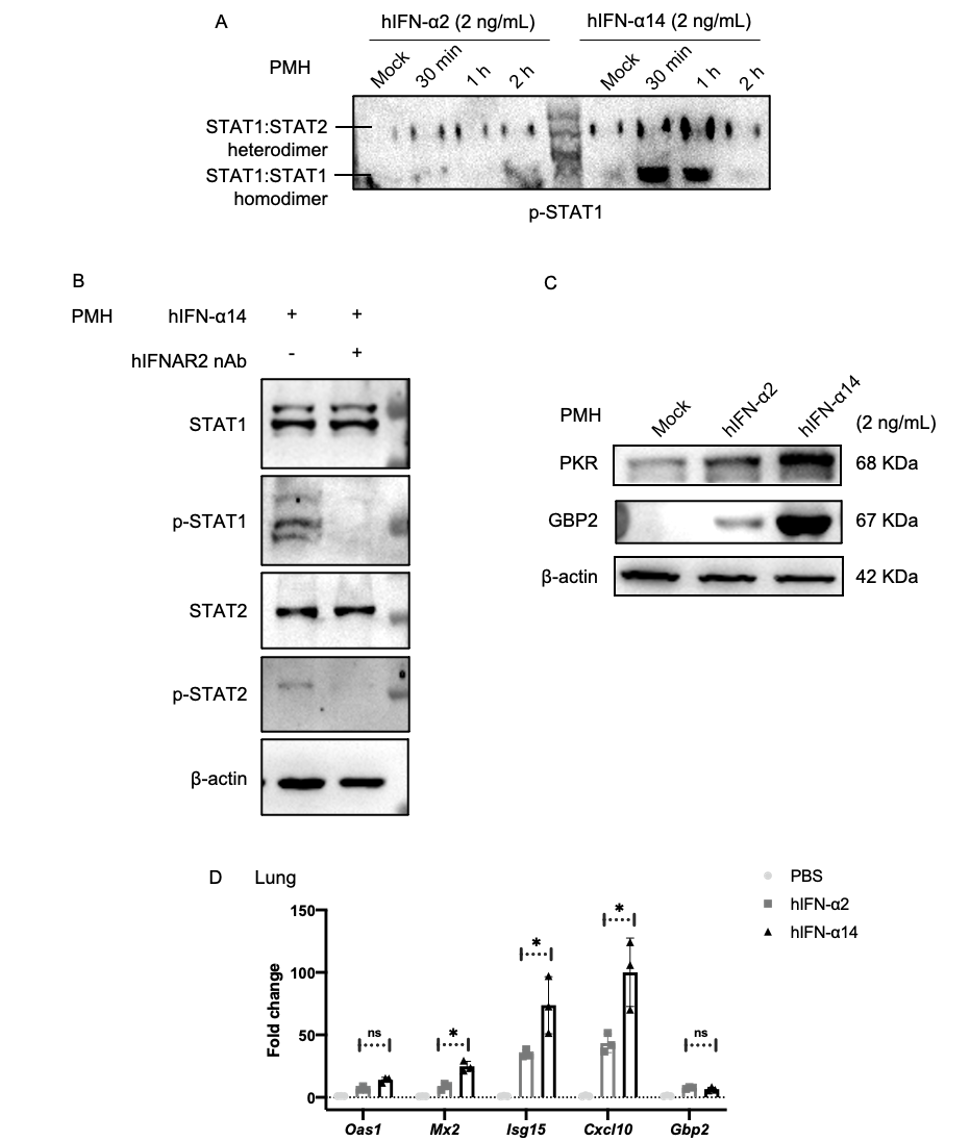
Figure S4. Effects of human IFN-α2 and -α14 subtypes on IFN-α and -γ signaling in IFNAR-hEC mice.** (A) Primary mouse hepatocytes were treated with 2 ng/mL of IFN-α2 or IFN-α14 for the indicated time points. The formation of STAT1/STAT2 heterodimers and STAT1 homodimers was assessed using native PAGE, followed by immunoblotting. (B) Primary mouse hepatocytes were pre-treated with an IFNAR2-neutralizing antibody (5 μg/mL) for 1 hour, followed by treatment with 2 ng/mL of human IFN-α14 for 30 minutes. The phosphorylation levels of STAT1 and STAT2 in the cells were examined by immunoblotting. (C) The expression of PKR and GBP2 at 24 h after 2 ng/mL hIFN-α2 or hIFN-α14 treatment was analyzed by immunoblotting. (D) *Ifnar*^hEC/+^ mice were intravenously administered hIFN-α2 or hIFN-α14 for 6 hours and subsequently sacrificed. Total RNA extracted from lung samples was subjected for RT-qPCR analysis of representative ISGs. Statistically significant differences are indicated by * for p < 0.05.


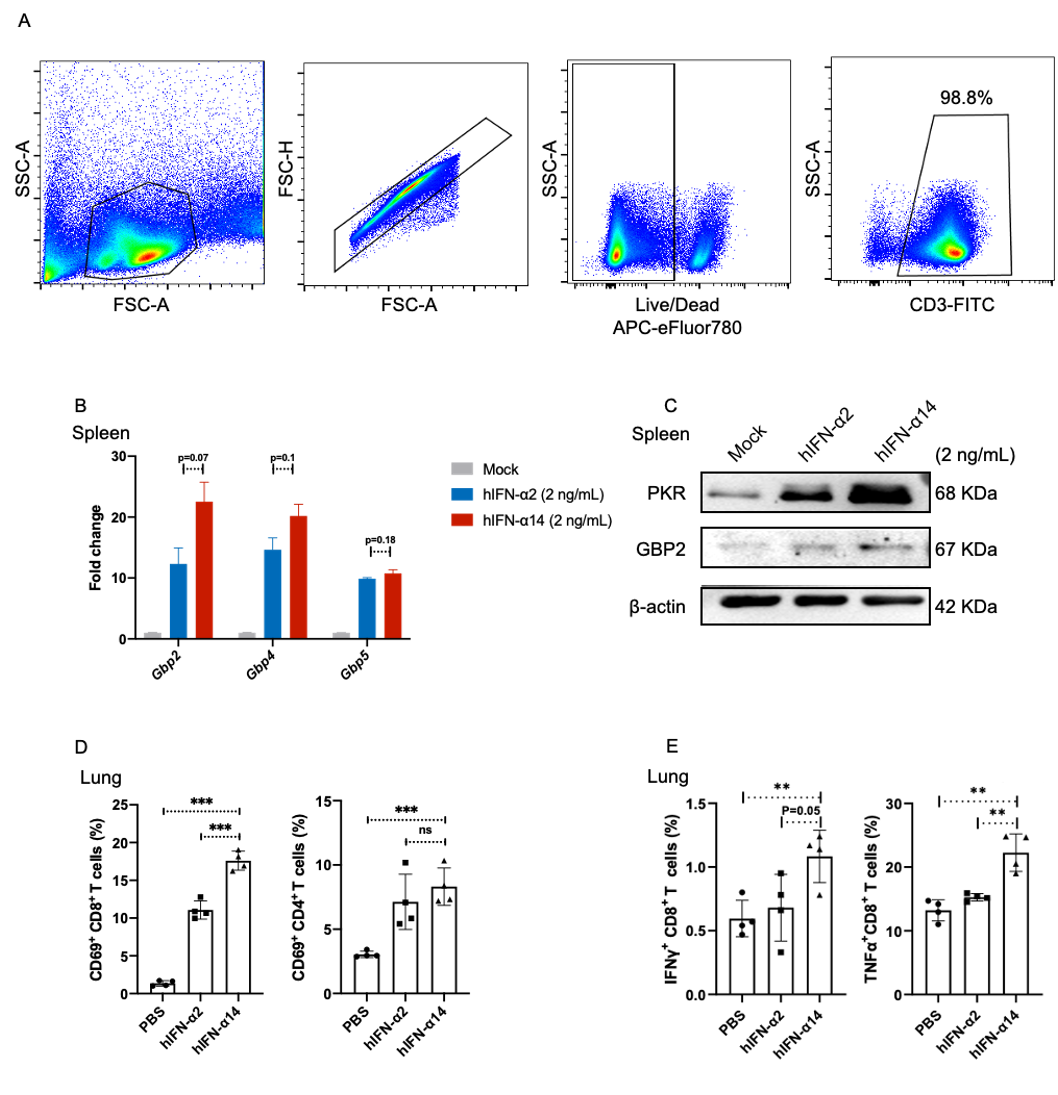


**Figure S5. The response of spleen and lung T cells in IFNAR-hEC mice to human IFN-α2 and IFN-α14.** (A) Single-cell suspensions of mouse spleen were used for positive selection of CD3+ T cells. Dead cells were excluded; cells were stained with anti-mouse CD3-FITC, then subjected to flow cytometry analysis. (B, C) Mouse spleen CD3+ T cells were enriched and treated with 2 ng/mL human IFN-α2 or IFN-α14 for 6 hours, and the mRNA levels of GBPs (B) were quantified by RT-qPCR. The expression of PKR and GBP2 (C) at 24 h after treatment was analyzed by immunoblotting. (D, E) *Ifnar*^hEC/+^ mice were intravenously administered hIFN-α2 or hIFN-α14 for 6 hours and subsequently, then sacrificed. The percentage of CD69^+^ T cells (D), and the percentage of IFN-γ or TNF-α producing CD8^+^ T cells (E) were analyzed by flow cytometry. Statistically significant differences are indicated by * for p < 0.05, ** for p < 0.01 and *** for p < 0.001.

**
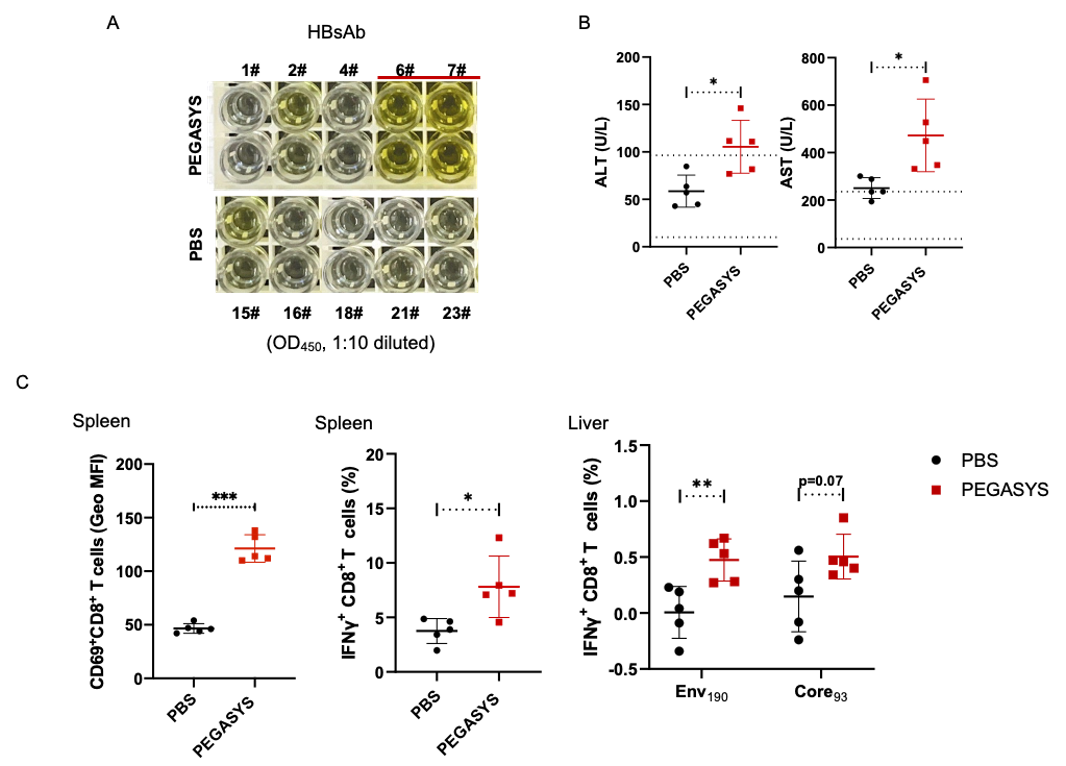
Figure S6. Antiviral and immunomodulatory activities of PEGASYS during HBV persistence.** (A) Mouse serum samples were qualitatively assayed for HBsAb by enzyme-linked immunosorbent assays. (B) The serum levels of AST and ALT at the endpoint were quantitated. (C) Mice were sacrificed after treatment with PEGASYS for 4 weeks, and the percentages of CD69^+^ CD8^+^ T cells, and IFN-γ-producing CD8+ T cells in both splenocytes and intrahepatic lymphocytes were measured by flow cytometry. Statistically significant differences are indicated by * for p < 0.05, ** for p < 0.01 and *** for p < 0.001. Abbreviations: ALT, alanine aminotransferase; AST, aspartate aminotransferase.
